# Concordance‐discordance between apolipoprotein B and lipid biomarkers in predicting 20‐year atherosclerotic cardiovascular disease risk: The ATTICA study (2002–2022)

**DOI:** 10.1111/eci.70077

**Published:** 2025-05-19

**Authors:** Sofia‐Panagiota Giannakopoulou, Smaragdi Antonopoulou, Fotios Barkas, Evangelos Liberopoulos, Christina Chrysohoou, Petros P. Sfikakis, Christos Pitsavos, Costas Tsioufis, Demosthenes Panagiotakos

**Affiliations:** ^1^ Department of Nutrition and Dietetics School of Health Sciences and Education, Harokopio University Athens Greece; ^2^ Department of Internal Medicine Medical School, University of Ioannina Ioannina Greece; ^3^ First Department of Propaedeutic and Internal Medicine Medical School, National and Kapodistrian University of Athens, Laiko General Hospital Athens Greece; ^4^ First Cardiology Clinic Medical School, National and Kapodistrian University of Athens, Hippokration Hospital Athens Greece

**Keywords:** apolipoprotein B, atherosclerotic cardiovascular disease risk, discordance, LDL‐C, lipoprotein(a), non‐HDL‐C

## Abstract

**Background:**

A strong correlation exists between low‐density lipoprotein cholesterol (LDL‐C), non‐high‐density lipoprotein cholesterol (non‐HDL‐C), and apolipoprotein B100 (apoB). However, evidence suggests that LDL‐C and non‐HDL‐C may underestimate apoB, potentially obscuring residual cardiovascular risk. Furthermore, interactions between apoB and lipoprotein(a) are implicated in atherogenesis. This study sought to determine whether discordance between apoB, LDL‐C, non‐HDL‐C, or lipoprotein(a) is associated with 20‐year atherosclerotic cardiovascular disease (ASCVD) risk within a cohort of apparently healthy adults.

**Methods:**

A cohort of 3042 CVD‐free adults residing in greater Athens, Greece, was recruited in 2002. A 20‐year follow‐up was conducted in 2022, comprising *n* = 2169 participants, of which *n* = 1988 had complete data for cardiovascular disease incidence. Discordance between biomarkers was defined based on recommended lipid thresholds. Cox proportional hazards models were used to assess the association between discordant/concordant biomarker pairs and 20‐year ASCVD risk.

**Results:**

ApoB strongly correlated with LDL‐C and non‐HDL‐C, though concordance was limited. Increased 20‐year ASCVD cumulative incidence with elevated apoB levels, beyond LDL‐C, non‐HDL‐C, and lipoprotein(a). Discordance analysis revealed that elevated apoB independently predicted increased 20‐year ASCVD risk, regardless of non‐HDL‐C and lipoprotein(a). However, this effect was observed only on concomitantly elevated LDL‐C levels. Incorporating apoB into the assessment of traditional modifiable risk factors elucidated part of the previously residual 20‐year ASCVD risk, especially in individuals with elevated LDL‐C, non‐HDL‐C, or lipoprotein(a) levels.

**Conclusions:**

ApoB may be a superior biomarker for assessing long‐term ASCVD risk, indicating that apoB‐containing lipoprotein particle number, rather than cholesterol content, is a more robust predictor of ASCVD risk.

## Graphical abstract

This study examined the association between 20‐year atherosclerotic cardiovascular disease (ASCVD) risk and discordance among apolipoprotein B100 (apoB) and lipid biomarkers, using established thresholds. ApoB strongly correlated with LDL‐C and non‐HDL‐C, though concordance was limited. ApoB independently associated with increased 20‐year ASCVD risk, regardless of non‐HDL‐C and lipoprotein(a). Incorporating apoB into the assessment of traditional modifiable risk factors elucidated part of the previously residual 20‐year ASCVD risk, suggesting ApoB may be a superior biomarker for assessing long‐term ASCVD risk.

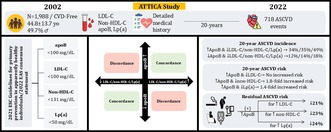

## 1 INTRODUCTION

Atherosclerotic cardiovascular disease (ASCVD) continues to be a major global health challenge, with elevated low‐density lipoprotein cholesterol (LDL‐C) recognized as a key causal factor.^1^ Therefore, LDL‐C forms the basis of various international guidelines for ASCVD prevention and treatment, as well as risk evaluation, playing a central role in routine clinical practice.^2^, ^3^ Structurally, LDL particles are composed of a cholesterol ester core, a lipogenic protein and apolipoprotein B (apoB), which facilitates binding to low‐density lipoprotein receptors (LDLRs) on cell membranes.^4^, ^5^ Beyond LDL particles, growing evidence suggests that other lipoprotein particles, such as very‐low‐density lipoprotein (VLDL) particles, are equally atherogenic.^6^ Apolipoprotein B (apoB) quantifies the total number of the atherogenic particles, such as LDL, VLDL, and lipoprotein(a) [Lp(a)].^6^ However, since LDL particles represent the predominant atherogenic lipoproteins in most individuals, LDL‐C and apoB levels are closely linked. As a result, elevated LDL‐C typically corresponds to elevated apoB, and vice versa. Because of this close correlation, demonstrating the additional value of apoB over LDL‐C in assessing ASCVD risk has been challenging.^7^

The 2019 European Atherosclerosis Society (EAS)/European Society of Cardiology (ESC) guidelines recognize apoB as a superior biomarker for cardiovascular risk assessment, particularly in populations where LDL‐C measurements may underestimate atherosclerotic risk. Given that apoB concentration serves as a direct metric of the total number of circulating atherogenic lipoprotein particles, apoB provides a comprehensive assessment of cardiovascular risk and a more accurate estimate of atherogenic burden, with standardized, accurate, and widely available measurement methods ensuring reliability for both risk assessment and treatment monitoring.^2^

Although apoB levels were traditionally considered a reliable correlate of calculated LDL‐C,^8^ recent research suggests that neither LDL‐C nor non‐HDL‐C fully represents apoB in assessing and managing an individual's clinical needs for better management.^9^ Moreover, evidence indicates a potential interaction between apoB and Lp(a) in the atherogenic process of Lp(a), thereby influencing ASCVD risk.^10^ Therefore, the primary aim of this study was to investigate the concordance/discordance between LDL‐C, non‐HDL‐C, Lp(a) and apoB levels, as well as to evaluate the incremental value of apoB on the risk of new onset ASCVD over 20 years.

## 2 METHODS

### 2.1 Study design

The ATTICA study is a prospective (2002–2022) epidemiological cohort with three follow‐up assessments. The study's objectives were to record the distribution of various socio‐demographic, lifestyle, clinical, biochemical, and psychological risk factors for ASCVD and to evaluate their predictive value in relation to long‐term ASCVD risk. More details about the study's objectives, design, sampling method, and methodology are available in previously published papers.^11^, ^12^

### 2.2 Setting and participants

The study participants were residents of the Attica region in Greece, with 78% coming from urban municipalities, including the capital city, Athens. The baseline sample consisted of 3042 individuals (45 ± 14 years, 49.7% males) out of 4056 who were invited to participate (75% participation rate), all free of ASCVD, cancer and other chronic inflammatory diseases, as evaluated by the physicians of the study. To mitigate selection bias, the sampling process was random and stratified by sex, age group, and region, in accordance with the 2001 census.

### 2.3 Bioethics

The ATTICA study complies with the ethical standards of the Declaration of Helsinki and has received approval by the Ethics Committee of the First Cardiology Department of the National and Kapodistrian University of Athens (#017/01.05.2001) and the Ethics Committee of the Harokopio University (#38/29.03.2022). Participants were informed about the aims and procedures and provided written consent to participate.

### 2.4 Biochemical measurements

Details about the ATTICA study socio‐demographic, clinical, and biochemical measurements and procedures followed on have been published elsewhere.^11^ Briefly, ApoB was measured by rate immunonephelometry. Lipoprotein (a) was quantified in mg/dL, with a latex‐enhanced turbidimetric immuno‐assay by Roche Diagnostics. Blood lipid profiles (total cholesterol, HDL‐C and triglycerides) were determined using a chromatographic enzymatic method in an automatic analyser RA‐1000 (Dade Behring, Marburg, Germany). HDL cholesterol was determined after precipitating apoB‐containing lipoproteins with dextran‐magnesium‐chloride. Serum for the measurement of these lipids was collected immediately after admission. LDL‐C was calculated using the Friedewald formula: {total cholesterol} – {HDL cholesterol} – 1/5 (triglycerides). Non‐HDL‐C was calculated by subtracting HDL‐C from total cholesterol. Intra‐ and inter‐assay coefficients of variation for total cholesterol, triglycerides, and HDL‐C did not exceed 3%, 4% and 4%, respectively. The intra‐ and inter‐assay coefficients of variation for Lp(a) were 2% and 5%, respectively.

### 2.5 Anthropometric measurements

Body weight and height were measured according to standardized protocols. Subsequently, the Body Mass Index (BMI) was calculated as the ratio of weight to height squared. Participants were categorized as overweight if their BMI fell within the range of 25–29.9 kg/m^2^ and as obese if their BMI was 30 kg/m^2^ or greater.^13^ Waist circumference (WC) and hip circumference (HC) were measured, and the waist‐to‐hip ratio (WHR) was subsequently calculated by dividing the waist measurement by the hip measurement. Established risk thresholds for elevated WHR were applied: >.90 for men and >.85 for women.^14^

### 2.6 Clinical characteristics

Hypertension was diagnosed if the systolic blood pressure reached 140 mmHg or higher, the diastolic blood pressure was 90 mmHg or greater, or if antihypertensive medication was being used. Type 2 diabetes was determined by a fasting blood glucose level of 126 mg/dL or higher, or the use of insulin or oral hypoglycaemic agents.

### 2.7 Lifestyle characteristics

Dietary habits were assessed using a validated semi‐quantitative food frequency questionnaire.^15^ Overall dietary quality was evaluated based on adherence to the Mediterranean diet, as quantified by the MedDietScore, which ranges from 0 to 55; higher values indicate greater adherence.^16^ Physical activity levels were determined using the short‐form International Physical Activity Questionnaire (IPAQ), validated for the Greek population.^17^, ^18^ Smoking status was categorized as follows: current smokers (≥1 cigarette per day), former smokers (quit smoking at least 1 year prior), and never smokers (no smoking history).

### 2.8 Follow‐up and endpoint

Comprehensive clinical data were obtained through in‐person interviews conducted by trained healthcare professionals and by reviewing medical records. Participants were contacted for follow‐up evaluations. For participants who died during the follow‐up period, information was gathered from family members and official death certificates. Specifically, in 2022, out of the initial 3042 participants, 2169 (71% participation rate) were located and provided their consent to participate in the follow‐up examination. Among those lost to follow‐up (i.e. 873 individuals), 771 could not be contacted due to inaccurate or outdated contact information, while 102 declined to participate in the screening without providing reasons. After excluding those with non‐complete ASCVD data at the 20‐year follow‐up (*n* = 181), the final analysed sample consisted of 1988 subjects (44.8 ± 13.7 years, 49.7% males). There were no clinically significant differences in age and sex distribution between this sub‐sample and the baseline group (*p*‐values >.05). The primary endpoint was the incidence of fatal or non‐fatal ASCVD events as classified by the World Health Organization (WHO) International Coding Diseases (ICD)‐10 standards.

### 2.9 Statistical analysis

The strength of association between lipid levels was assessed using Spearman's rank correlation coefficient. To investigate the concordance between LDL‐C, non‐HDL‐C and Lp(a) with apoB levels, the cohort was stratified into quintiles for each parameter using a previously described methodology.^19^ A priori, we defined agreement as the placement of a subject in the same quintile for both LDL‐C, non‐HDL‐C or Lp(a) and apoB. Under this criterion, concordance would imply identical distributions across quintiles for both parameters. Conversely, discordance would be indicated by a subject's ranking in different quintiles for the two parameters. To quantify the overall agreement, a contingency table analysis was performed, and Cohen's kappa statistic was calculated. A kappa value of 1 represents perfect agreement, 0 indicates agreement equivalent to chance, and a negative value suggests agreement worse than chance.^20^ Participants were initially categorized based on LDL‐C levels, utilizing the 100 mg/dL threshold recommended by the European Society of Cardiology (ESC) Guidelines for primary prevention in apparently healthy individuals.^8^ Corresponding thresholds for non‐HDL‐C and apoB were 131 mg/dL and 100 mg/dL, respectively. The 2022 EAS consensus statement's threshold of 50 mg/dL for Lp(a) was used to group participants as being at increased risk.^21^ Subsequently, participants were classified based on the discordance observed between apoB levels and either LDL‐C, non‐HDL‐C, or Lp(a) levels. Continuous variables were evaluated for normality using P–P plots and are presented as mean values with standard deviations for the normally distributed and as median values with interquartile ranges (IQR) for the non‐normally distributed. Categorical variables are expressed as frequencies. The chi‐squared test was used to examine the associations between categorical variables. The one‐way analysis of variance (ANOVA) was used to compare the mean values of normally distributed variables. For non‐normally distributed continuous variables, median values were compared using the non‐parametric K‐sample test on the equality of medians with continuity correction. The cumulative incidence of ASCVD was calculated by dividing the number of new cases by the total number of participants in the 20‐year follow‐up study. Cox proportional hazards models were employed to assess whether individuals with discordant or concordant apoB and non‐HDL‐C/LDL‐C/Lp(a) values exhibited elevated 20‐year ASCVD risk, stratified into groups according to the ESC threshold values for apoB, LDL‐C and non‐HDL‐C and EAS threshold for Lp(a). Results are reported as hazard ratios (HRs) and their respective 95% confidence intervals (CIs). Analyses were conducted adjusted for a range of sociodemographic (e.g. age and sex), lifestyle (e.g. smoking status and physical activity) and clinical factors (e.g. hypertension and diabetes). A standard modifiable cardiovascular risk factors (SMuRFs) score for ASCVD was calculated based on prevailed baseline hypertension, hypercholesterolemia, diabetes, abnormal WHR (based on established thresholds), and smoking status (ever/never), resulting in a range of 0–5. Stratified analyses were performed by the number of SMuRFs. Population Attributable fraction (PAF) was calculated using a methodology suggested by others.^22^, ^23^ All analyses were performed using STATA 18 (Stata‐Corp, College Station, TX). Statistical hypotheses were 2‐tailed and a *p*‐value <.05 was considered as the level of significance.

## 3 RESULTS

### 3.1 Concordance‐Discordance between apoB, LDL‐C, non‐HDL‐C and Lp(a)

ApoB exhibited a strong positive correlation with LDL‐C (Spearman's rho = .712, *p* < .001) and non‐HDL‐C (Spearman's rho = .794, *p* < .001), and a fair positive correlation with Lp(a) (Spearman's rho = .145, *p* < .001). Table 1 demonstrates concordance/discordance between lipid measurements. Overall, approximately 48.7% of LDL‐C and apoB values were concordant, with higher concordance (~60%) observed in the extreme quintiles compared to intermediate ranges. Quintile classification yielded a kappa of .357 (fair agreement). Non‐HDL‐C and apoB exhibited a higher level of concordance (58.3%) yielding a kappa of .477, indicating moderate agreement. However, apoB and Lp(a) showed negligible concordance (Cohen kappa = .045).

**TABLE 1:** Concordance analysis by quintiles of apolipoprotein B (apoB) and low‐density lipoprotein cholesterol (LDL‐C), non‐high‐density lipoprotein cholesterol (non‐HDL‐C) and lipoprotein (a) (Lp(a)) levels of ATTICA study participants.

	Quintiles of ApoB (mg/dL)				
	.5–80.6	80.7–96.9	97.0–113.0	114.0–132.0	133.0–316.0
Quintiles of LDL‐C (mg/dL)					
22.0–90.5	66.0^a^	18.5	6.5	4.3	3.8
90.5–110.2	22.8	41.3^a^	20.5	7.9	5.4
110.2–127.9	3.8	28.6	38.1^a^	21.3	8.5
127.9–151.2	3.8	8.3	27.0	39.3^a^	23.3
151.2–317.5	3.6	3.3	7.9	27.2	59.0^a^
Cohen's κ	.358 (*p* < .001)				
Quintiles of non‐HDL‐C (mg/dL)					
32.0–108.7	74.5^a^	18.7	2.4	2.0	1.9
108.9–131.1	16.1	51.3^a^	21.2	4.9	3.3
131.1–152.4	3.1	21.8	47.1^a^	20.6	5.1
152.5–179.6	2.5	5.8	24.1	49.8^a^	21.1
179.7–374.8	3.8	2.4	5.2	22.7	68.6^a^
Cohen's κ	.477 (*p* < .001)				
Quintiles of Lp(a) (mg/dL)					
.92–3.75	28.1^a^	18.7	16.4	18.4	19.6
3.76–7.99	22.2	23.2^a^	19.2	15.9	16.3
8.01–14.3	20.3	20.9	20.7^a^	19.7	17.2
14.4–28.5	16.7	17.6	24.7	20.3^a^	21.1
28.6–216	12.7	19.6	19.0	25.7	25.8^a^
Cohen's κ	.045 (*p* < .001)				

^a^ Concordant, that is, the proportion of subjects in whom atherogenic indexes fell into the same quintile of the distribution.

In Tables 2, 3, 4 the baseline risk factor profiles associated with apoB/LDL‐C, apoB/non‐HDL‐C, and apoB/Lp(a) discordance are presented, respectively. Individuals with apoB levels exceeding the thresholds, irrespective of LDL‐C, non‐HDL‐C or Lp(a) concentration, demonstrated a distinct risk factor profile compared to those below the threshold. This group was predominantly male, older, with lower levels of physical activity, poorer adherence to a Mediterranean diet, and higher rates of obesity, diabetes and hypertension. Furthermore, these individuals had lower HDL‐C levels and elevated triglyceride levels, indicative of a phenotype characterized by small‐dense lipoprotein particles. The observed differences in risk factor profiles were more pronounced in cases of apoB discordance with LDL‐C and Lp(a) compared to discordance with non‐HDL‐C.

**TABLE 2:** Baseline characteristics of ATTICA study participants stratified by apolipoprotein B/low‐density lipoprotein cholesterol (LDL‐C) levels (apoB/LDL‐C) categories defined by European Society of Cardiology (ESC) thresholds.

	ApoB and LDL‐C <100 mg/dL (*n* = 540) (24%)	ApoB ≥100 mg/dL, LDL‐C <100 mg/dL (*n* = 100) (5%)	ApoB <100 mg/dL, LDL‐C ≥100 mg/dL (*n* = 437) (20%)	ApoB and LDL‐C ≥100 mg/dL (*n* = 1131) (51%)	*p*‐Value
Demographic and lifestyle					
Age (years), mean ± SD	36 (12)	46 (15)	42 (13)	49 (12)	<.001
Sex (% males)	39	59	38	57	<.001
Smoking (%)	43	49	40	45	.227
Physical activity (% active)	45	30	44	39	.008
MedDietScore (0–55), mean ± SD	29 (7)	25 (6)	28 (7)	25 (6)	<.001
Clinical factors					
BMI (kg/m^2^), mean ± SD	24.5 (4.1)	27.1 (4.3)	25.0 (4.2)	27.4 (4.5)	<.001
Hypertension (%)	16	32	22	40	<.001
Diabetes (%)	3	10	4	8	<.001
Family History CVD (%)	34	35	39	37	.724
Obesity (%)	10	23	13	24	<.001
Biochemical factors					
HDL‐C (mg/dL), mean ± SD	52.0 (14.9)	47.8 (16.1)	49.3 (12.5)	45.6 (11.4)	<.001
LDL‐C (mg/dL), mean ± SD	80.3 (14.9)	84.5 (15.8)	121.2 (22.3)	145.6 (29.6)	<.001
TGs (mg/dL), median (IQR)	65.5 (42.5)	148.5 (143)	76 (41)	120 (76)	<.001
Non‐HDL‐C (mg/dL), mean ± SD	96.1 (17.7)	123.9 (33.7)	138.5 (25.8)	173.1 (32.7)	<.001
ApoB (mg/dL), mean ± SD	73.9 (13.1)	122.1 (23.4)	87.1 (13.5)	128.3 (20.8)	<.001
Lp(a) (mg/dL), median (IQR)	8.1 (13.7)	10.3 (21.9)	10.6 (18.6)	13.1 (22.2)	<.001

*Note*: Continuous variables are presented as mean values (±standard deviation) for the normally distributed and median (inter‐quartile‐range, IQR) for the rest, while categorical variables are presented as relative frequencies (percentages). *p*‐values referring to differences between the groups are derived using the chi‐square test for the categorical variables, one‐way ANOVA for the normally distributed continuous variables, and the nonparametric K‐sample test on the equality of medians with continuity correction for the rest.

Abbreviations: BMI, body‐mass index; HDL‐C, high‐density lipoprotein cholesterol; LDL‐C, low‐density lipoprotein cholesterol (calculated via Friedewald equation); TGs, triglycerides.

**TABLE 3:** Baseline characteristics of ATTICA study participants stratified by apolipoprotein B/non‐high‐density lipoprotein cholesterol (non‐HDL‐C) levels (apoB/non‐HDL‐C) categories defined by European Society of Cardiology (ESC) thresholds.

	ApoB <100 mg/dL, non‐HDL‐C <131 mg/dL (*n* = 749) (34%)	ApoB ≥100 mg/dL, non‐HDL‐C <131 mg/dL (*n* = 126) (6%)	ApoB <100 mg/dL, non‐HDL‐C ≥131 mg/dL (*n* = 230) (10%)	ApoB ≥100 mg/dL, non‐HDL‐C ≥131 mg/dL (*n* = 1113) (50%)	*p*‐Value
Demographic and lifestyle					
Age (years), mean ± SD	37 (12)	45 (15)	45 (14)	49 (12)	<.001
Sex (% males)	38	52	38	58	<.001
Smoking (%)	44	43	35	46	.037
Physical activity (% active)	46	34	41	39	.011
MedDietScore (0–55), mean ± SD	29 (7)	26 (6)	27 (6)	25 (6)	<.001
Clinical factors					
BMI (kg/m^2^), mean ± SD	24.5 (4.0)	26.4 (4.5)	25.5 (4.4)	27.5 (4.5)	<.001
Hypertension (%)	17	28	24	40	<.001
Diabetes (%)	3	6	5	9	<.001
Family History CVD (%)	34	32	44	37	.192
Obesity (%)	10	20	16	25	<.001
Biochemical factors					
HDL‐C (mg/dL), mean ± SD	51.8 (14.2)	49.7 (13.1)	47.6 (12.7)	45.3 (11.6)	<.001
LDL‐C (mg/dL), mean ± SD	88.2 (17.9)	96.1 (15.4)	132.2 (26.6)	145.7 (30.8)	<.001
TGs (mg/dL), median (IQR)	67 (36)	94 (51)	93 (64)	125 (82)	<.001
Non‐HDL‐C (mg/dL), mean ± SD	103.1 (18.7)	116.2 (15.9)	154.1 (27.4)	175.3 (31.8)	<.001
ApoB (mg/dL), mean ± SD	77.6 (13.4)	119.4 (23.6)	87.3 (16.4)	128.9 (20.6)	<.001
Lp(a) (mg/dL), median (IQR)	9.0 (15.1)	15.6 (24.4)	10.5 (18.7)	12.5 (22.0)	<.001

*Note*: Continuous variables are presented as mean values (± standard deviation) for the normally distributed and median (inter‐quartile‐range, IQR) for the rest, while categorical variables are presented as relative frequencies (percentages). *p*‐values referring to differences between the groups are derived using the chi‐square test for the categorical variables, one‐way ANOVA for the normally distributed continuous variables, and nonparametric K‐sample test on the equality of medians with continuity correction for the rest.

Abbreviations: BMI, body‐mass index; HDL‐C, high‐density lipoprotein cholesterol; LDL‐C, low‐density lipoprotein cholesterol (calculated via Friedewald equation); TGs, triglycerides.

**TABLE 4:** Baseline characteristics of ATTICA study participants stratified by apolipoprotein B/lipoprotein (a) (Lp(a)) levels (apoB/Lp(a)) discordance categories defined by European Society of Cardiology/ European Atherosclerosis Society (ESC/EAS) thresholds.

	ApoB <100 mg/dL, Lp(a) <50 mg/dL (*n* = 971) (41%)	ApoB ≥100 mg/dL, Lp(a) <50 mg/dL (*n* = 1177) (50%)	ApoB <100 mg/dL, Lp(a) ≥50 mg/dL (*n* = 59) (3%)	ApoB ≥100 mg/dL, Lp(a) ≥50 mg/dL (*n* = 142) (6%)	*p*‐Value
Demographic and lifestyle					
Age (years), mean ± SD	39 (13)	48 (13)	37 (13)	50 (13)	<.001
Sex (% males)	37	57	32	51	<.001
Smoking (%)	41	46	39	42	.214
Physical activity (% active)	45	39	46	38	.013
MedDietScore (0–55), mean ± SD	28 (7)	25 (6)	27 (6)	24 (5)	<.001
Clinical factors					
BMI (kg/m^2^), mean ± SD	24.8 (4.2)	27.4 (4.5)	24.1 (3.9)	27.0 (4.2)	<.001
Hypertension (%)	19	39	21	35	<.001
Diabetes (%)	3	9	5	6	<.001
Family History CVD (%)	37	35	23	41	.485
Obesity (%)	11	24	5	18	<.001
Biochemical factors					
HDL‐C (mg/dL), mean ± SD	50.7 (13.9)	45.5 (11.7)	52.8 (14.5)	47.7 (12.6)	<.001
LDL‐C (mg/dL), mean ± SD	98.4 (27.2)	140.8 (33.0)	103.1 (34.1)	141.9 (34.6)	<.001
TGs (mg/dL), median (IQR)	71 (42)	123 (81)	69 (41)	115 (76)	<.001
Non‐HDL‐C (mg/dL), mean ± SD	114.9 (29.9)	169.7 (35.4)	119.8 (35.9)	167.9 (36.4)	<.001
ApoB (mg/dL), mean ± SD	79.6 (14.8)	128.0 (21.7)	81.5 (18.1)	130.8 (22.5)	<.001
Lp(a) (mg/dL), median (IQR)	8.4 (12.8)	11.0 (15.9)	67.0 (24.8)	75.1 (38.9)	<.001

*Note*: Continuous variables are presented as mean values (±standard deviation) for the normally distributed and median (inter‐quartile‐range, IQR) for the rest, while categorical as relative frequencies (percentages). P‐values referring to differences between the groups, derived using the chi‐square test for the categorical variables, one‐way ANOVA for the normally distributed continuous variables and nonparametric K‐sample test on the equality of medians with continuity correction for the rest.

Abbreviations: BMI: body‐mass index; HDL‐C: high‐density lipoprotein cholesterol; LDL‐C: low‐density lipoprotein cholesterol (calculated via Friedewald equation); TGs: triglycerides.

### 3.2 ASCVD incidence and mortality at 20‐year follow‐up

Over the 20‐year follow‐up period, 718 participants (40% of males and 32% of females; *p* < .001) experienced an adverse ASCVD event (fatal or non‐fatal). Among these, 96 individuals had a fatal event (mortality rate: 7.3% in males and 1.8% in females; *p* < .001). A detailed analysis of lifestyle and clinical characteristics of the participants throughout the 20‐year follow‐up period has been published elsewhere.^12^

**FIGURE 1:**
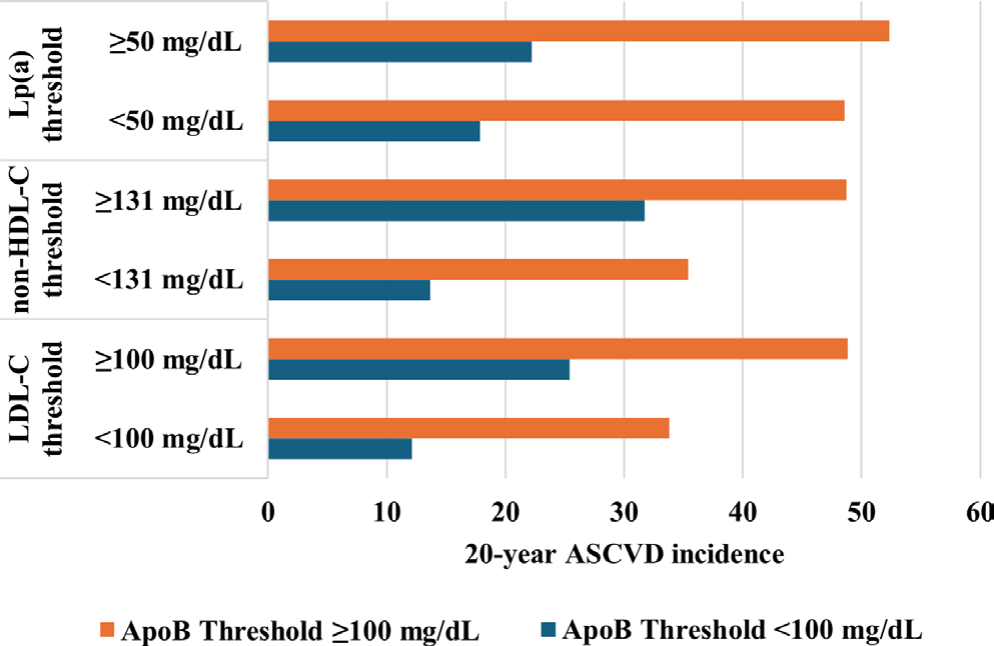
Cumulative incidence of atherosclerotic cardiovascular disease (ASCVD) events over a 20‐year period, stratified by low‐density lipoprotein cholesterol (LDL‐C), non‐high‐density lipoprotein cholesterol (non‐HDL‐C) and lipoprotein(a) (Lp(a)) thresholds, with further stratification by apolipoprotein B (apoB) levels.

In Figure 1 the cumulative incidence of ASCVD over the 20‐year period, stratified by apoB levels utilizing guideline‐recommended thresholds, is presented. Irrespective of LDL‐C, non‐HDL‐C, or Lp(a) levels, individuals categorized as having high apoB levels consistently exhibited elevated ASCVD rates.

### 3.3 Discordant lipid traits and 20‐year ASCVD risk

Both continuous apoB levels (HR per 1 mg/dL: 1.006, 95% CI: 1.002–1.010, *p* = .005) and apoB levels exceeding the threshold of 100 mg/dL (HR 1.431, 95% CI 1.106–1.852, *p* = .006) were significantly associated with increased risk of ASCVD over 20 years, after multivariable adjustment for demographic (age, sex), lifestyle (BMI, smoking status, physical activity, Mediterranean Diet adherence), and clinical factors (hypertension, diabetes, triglyceride levels, HDL‐C levels, family history of ASCVD).

**FIGURE 2:**
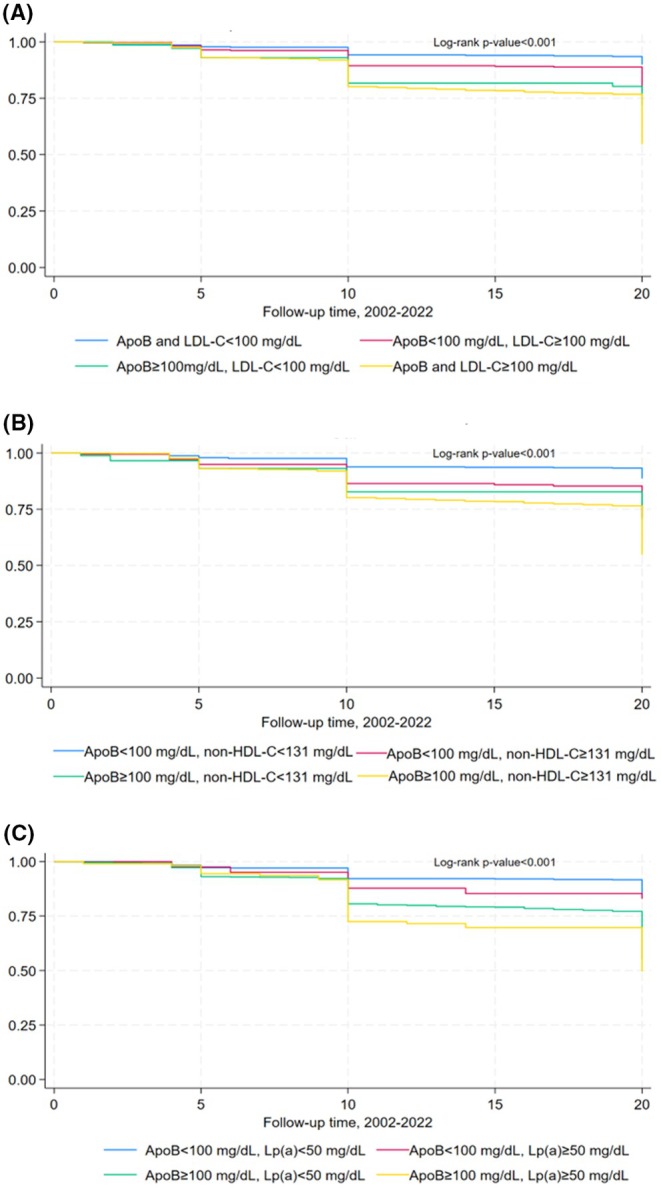
Kaplan–Meier survival curves for the ATTICA study sample, during the 20‐year follow‐up period, 2002–2022, stratified by (A) apolipoprotein B/low‐density lipoprotein cholesterol (LDL‐C) levels (apoB/LDL‐C), (B) apolipoprotein B/non‐high‐density lipoprotein cholesterol (non‐HDL‐C) levels (apoB/non‐HDL‐C), (C) apolipoprotein B/lipoprotein (a) (Lp(a)) levels (apoB/Lp(a)).

Individuals with discordant apoB levels exceeding established thresholds, coupled with non‐HDL or Lp(a) levels below their respective thresholds, presented a significantly increased risk for ASCVD (HR 1.794, 95% CI: 1.056–3.047, *p* = .031; HR 1.433, 95% CI: 1.099–1.867, *p* = .008, respectively), in comparison to those with concordant levels below the defined thresholds, following multivariable adjustment. In contrast, discordant apoB levels above the threshold with LDL‐C levels below were not associated with an elevated risk of ASCVD (Table 5). Figure 2 illustrates the Kaplan–Meier survival rate of the study's participants throughout the 20‐year follow‐up period, stratified by discordant groups.

**TABLE 5:** Results from Cox proportional hazards models exploring the association between baseline discordant and concordant categories of apolipoprotein B (apoB), low‐density lipoprotein cholesterol (LDL‐C) non‐high‐density lipoprotein cholesterol (non‐HDL‐C) and lipoprotein(a) (Lp(a)) levels based on European Society of Cardiology/ European Atherosclerosis Society (ESC/EAS) thresholds and the risk of developing an atherosclerotic cardiovascular (ASCVD) event throughout the 20‐year study period.

	Hazard ratio (95% CI)
ApoB (per 1 mg/dL)	1.006 (1.002–1.010)
LDL‐C (per 1 mg/dL)	1.006 (1.004–1.009)
Non‐HDL‐C (per 1 mg/dL)	1.006 (1.003–1.009)
Lp(a) (per 1 mg/dL)	1.003 (.999–1.007)
Elevated ApoB (ESC threshold)	
ApoB <100 mg/dL	Reference
ApoB ≥100 mg/dL	1.431 (1.106–1.852)
ApoB/LDL‐C concordance	
ApoB and LDL‐C <100 mg/dL	Reference
ApoB <100 mg/dL, LDL‐C ≥100 mg/dL	1.451 (.907–2.323)
ApoB ≥100 mg/dL, LDL‐C <100 mg/dL	1.320 (.665–2.620)
ApoB and LDL‐C ≥100 mg/dL	1.852 (1.220–2.811)
ApoB/non‐HDL‐C concordance	
ApoB <100 mg/dL, non‐HDL‐C <131 mg/dL	Reference
ApoB <100 mg/dL, non‐HDL‐C ≥131 mg/dL	1.411 (.907–2.193)
ApoB ≥100 mg/dL, non‐HDL‐C <131 mg/dL	1.794 (1.056–3.047)
ApoB ≥100 mg/dL, non‐HDL‐C ≥131 mg/dL	1.658 (1.184–2.322)
ApoB/Lp(a) concordance	
ApoB <100 mg/dL, Lp(a) <50 mg/dL	Reference
ApoB <100 mg/dL, Lp(a) ≥50 mg/dL	1.891 (.592–6.039)
ApoB ≥100 mg/dL, Lp(a) <50 mg/dL	1.433 (1.099–1.867)
ApoB ≥100 mg/dL, Lp(a) ≥50 mg/dL	1.612 (1.075–2.418)

*Note*: The reported HR have been derived after multivariable adjustment for age (continuous), sex, smoking status, MedDietScore, physical activity status, body mass index (BMI), triglyceride levels, high density lipoprotein (HDL) cholesterol levels, history of hypertension, diabetes and family history of CVD.

### 3.4 Subgroup analyses

Stratification by WHR thresholds revealed that the significant association between apoB levels and ASCVD risk was exclusive to those with WHR above the threshold (HR 1.005, 95% CI 1.000–1.011, *p* = .048). Similarly, when stratified by baseline SMuRFs number to assess its moderating effect on 20‐year ASCVD risk, the significant apoB‐ASCVD association was maintained only in individuals with two or more SMuRFs (HR 1.005, 95% CI 1.001–1.010, *p* = .019), after various adjustments.

### 3.5 Population‐attributable fractions (PAF) and residual risk of the 20‐year ASCVD incidence

The PAF of 20‐year ASCVD incidence was 20.3% (95% CI, 9.3–29.9) for apoB levels exceeding 100 mg/dL and 21.3% (95% CI, 11.7–28.9) for traditional modifiable risk factors (smoking, physical inactivity, hypertension, diabetes, obesity). When considering both elevated apoB levels and traditional modifiable risk factors, the PAF reached 37.2% (95% CI, 25.3 to 47.1). This suggests that incorporating apoB into the assessment of traditional risk factors elucidated an additional 20% of the previously residual 20‐year ASCVD risk.

**FIGURE 3:**
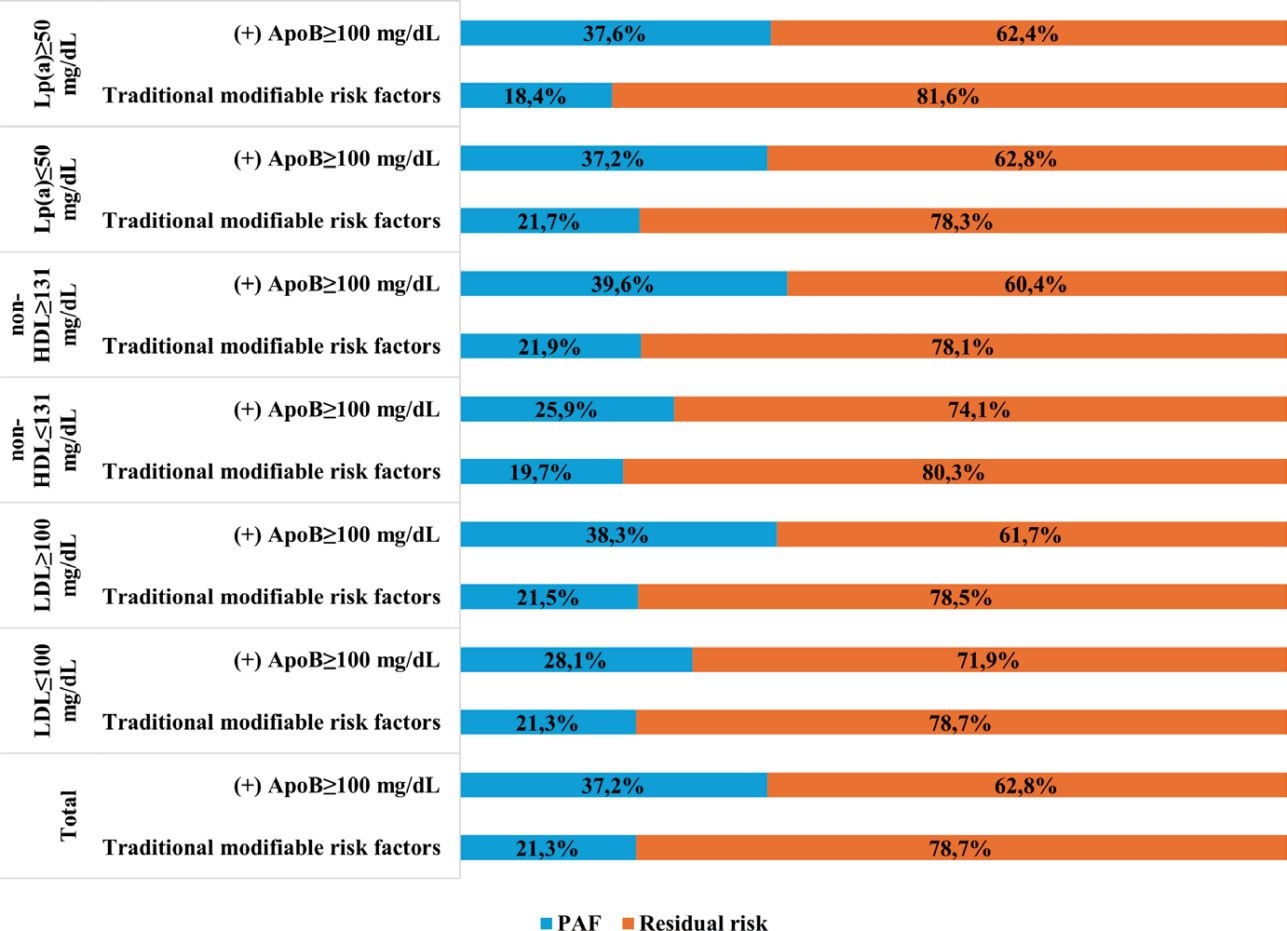
Population‐attributable fractions (PAF) and residual risk of the 20‐year incidence of atherosclerotic cardiovascular disease (ASCVD) for traditional modifiable risk factors (smoking, physical inactivity, hypertension, diabetes, obesity), for elevated apolipoprotein B (apoB) levels (≥100 mg/dL), stratified by low‐density lipoprotein cholesterol (LDL‐C), non‐high‐density lipoprotein cholesterol (non‐HDL‐C) and lipoprotein(a) (Lp(a)) levels.

Stratification by LDL‐C, non‐HDL‐C or Lp(a) levels demonstrated that incorporating apoB into the assessment of conventional risk factors significantly improved the elucidation of residual 20‐year ASCVD risk, particularly among individuals with elevated LDL‐C, non‐HDL‐C and Lp(a) levels (21%, 23% and 24% respectively) (Figure 3).

## 4 DISCUSSION

The present study revealed that, while a strong correlation exists between apoB and LDL‐C and non‐HDL‐C levels, the concordance among these parameters was only fair for LDL‐C and moderate for non‐HDL‐C. This finding aligns with prior observations^19^ and highlights the substantial influence of variations in the cholesterol content of apoB‐containing particles on the discrepancies observed between LDL‐C, non‐HDL‐C and apoB levels. Given this discordance, LDL‐C and non‐HDL‐C may not always serve as reliable surrogates for apoB. Therefore, in routine clinical practice, it is crucial to recognize potential discordance in individual patients. When observed, apoB should be considered as an additional therapeutic target, especially in treated patients, due to the generally larger relative reduction in LDL‐C compared to apoB.^24^ Furthermore, it was observed that elevated apoB levels were associated with higher 20‐year ASCVD cumulative incidence, independent of LDL‐C, non‐HDL‐C or Lp(a) levels, highlighting the limitations of current lipid profile analyses in accurately assessing ASCVD risk.

The definition of discordance is commonly approached through three distinct methodologies: cut‐point‐based, percentile‐based, and residual‐based analyses.^25^ Among these, the cut‐point‐based method is frequently regarded as the most intuitive and clinically applicable, as it allows for the categorization of individuals into concordant and discordant groups using various definitional thresholds, such as medians, tertiles, or quintiles.^25^ While studies employing these methodologies have demonstrated a degree of consistency in their findings,^26^, ^27^, ^28^, ^29^ it is important to acknowledge that the cut‐points utilized often represent arbitrary divisions derived from the distribution of the characteristic within the specific study population. On the other hand, residual analysis employed by some studies calculates and compares deviations from the regression line relating two markers, offering the least arbitrary and theoretically most informative approach,^9^, ^30^ it is more difficult to determine in clinical practice.^27^ This study defined discordance based on established thresholds from the 2021 ESC Guidelines on ASCVD prevention and the 2022 EAS consensus statement on Lp(a) to address the limitations of arbitrary definitions and enhance clinical relevance.

Our findings corroborate previous research^9^, ^10^ by showing that elevated apoB is associated with increased 20‐year ASCVD cumulative incidence, independently of LDL‐C, non‐HDL‐C and Lp(a) levels. Moreover, discordant analyses revealed that exceeding the apoB threshold significantly elevated long‐term ASCVD risk, regardless of non‐HDL‐C or Lp(a) levels, even after adjusting for lipid and non‐lipid factors. This aligns with INTERHEART findings of increased myocardial infarction risk when apoB exceeds non‐HDL‐C,^26^ and ARIC findings that demonstrate higher ASCVD and CHD risk in individuals with low Lp(a) and high apoB.^10^ Furthermore, in statin‐treated patients, discordance analysis reveals apoB's superior accuracy over LDL‐C and non‐HDL‐C as a marker for all‐cause mortality risk, and additionally highlights its enhanced accuracy compared to LDL‐C in predicting myocardial infarction risk.^31^

It was also observed in the present study that an increased long‐term ASCVD risk was exclusively associated with the co‐occurrence of elevated apoB and LDL‐C levels. This observation diverges from the findings of the Women's Health Study, which reported an elevated risk of incident coronary heart disease over a 17‐year period among women with LDL‐C levels below the median and apoB levels exceeding the median.^29^ However, the Quebec Cardiovascular Study demonstrated that the 5‐year risk of coronary artery disease in individuals with disproportionately elevated apoB levels was comparable to that observed in individuals with disproportionately elevated LDL‐C levels.^19^ These discrepancies may stem from differences in study populations or variations in the definition of “elevated” levels.

The primary determinant of cholesterol mass within apoB‐containing lipoprotein particles is the exchange of core lipids, specifically cholesteryl esters and triglycerides. This exchange occurs predominantly through the action of cholesteryl ester transfer protein (CETP), which facilitates the transfer of these lipids between apoB‐containing particles and HDL. These well‐documented processes explain the observed discrepancies between apoB levels and traditional cholesterol markers.^32^ Consistent with previous findings,^10^, ^30^ this study demonstrates that individuals with apoB levels exceeding a defined threshold, regardless of LDL‐C, non‐HDL‐C, or Lp(a) concentrations – exhibit a distinct risk factor profile, characterised by elevated triglycerides, reduced HDL‐C levels, and a significantly increased prevalence of hypertension, obesity, and diabetes.

The present study revealed a significant independent association between apoB levels and the 20‐year incidence of ASCVD, that upon stratification remained significant only in individuals with a WHR exceeding established risk thresholds, suggesting a potential moderating effect of central adiposity on the relationship between apoB and long‐term cardiovascular risk. It is increasingly recognized that the distribution of adipose tissue and the imbalance among various adipocytokines released therefrom are critical determinants of coronary circulatory function.^33^ The “obesity paradox” affecting coronary endothelial function, as highlighted in recent studies, indicates that the relationship between obesity and coronary microvascular function appears to exhibit a U‐shaped curve. This “U‐turn” phenomenon has been linked to complex alterations in adipocytokine profiles, inflammation, lipid and glucose metabolism,^34^ and likely reflects the contrasting effects of abdominal versus subcutaneous adipose tissue on coronary circulatory function, potentially representing distinct pathophysiological pathways.^33^

Data analysis revealed that 20% of incident ASCVD events over the 20‐year period were attributable to apoB levels exceeding 100 mg/dL. ΑpoB levels, along with traditional modifiable risk factors, explained nearly 40% of cases, elucidating an additional 20% of previously unexplained 20‐year ASCVD risk. This effect was pronounced among individuals with elevated LDL‐C, non‐HDL‐C or Lp(a) levels. These findings suggest that a combined approach including apoB‐lowering therapies alongside the management of traditional modifiable risk factors may yield a more substantial reduction in ASCVD risk compared to managing modifiable risk factors alone, especially in individuals with elevated LDL‐C, non‐HDL‐C or Lp(a) levels.

### 4.1 Strengths and Limitations

This study has several strengths. To our knowledge, the ATTICA study stands as one of the very few large‐scale prospective cohort studies on ASCVD epidemiology worldwide. The study cohort adequately represented the age and sex distribution of the urban referent population through 3 follow‐up examinations. A comprehensive evaluation of participants was undertaken, including clinical, biochemical, and lifestyle CVD factors at baseline. This study conducted a comprehensive investigation into the association between discordant and concordant categories of apoB, non‐HDL‐C, LDL‐C and Lp(a) and the incidence of ASCVD over a 20‐year follow‐up period, adjusted for a range of established risk factors. Furthermore, the study evaluated the extent to which incorporating apoB measurements into the assessment of traditional risk factors could further explain residual ASCVD risk. To the best of our knowledge, this area of research has been understudied in literature.

However, this study also has several limitations. Firstly, the analysis was based on baseline measurements, potentially leading to misclassification of transitions due to the prolonged intervals between follow‐up assessments. Secondly, the use of lipid‐lowering medication by the participants at baseline examination was low (10.8%), hindering stratification analysis. However, considering the limited prevalence of statin usage in our cohort, we believe that any potential bias arising from this factor is unlikely to substantially influence our study findings. Thirdly, the study population comprised individuals with generally low ASCVD risk. Therefore, the clinical applicability of our findings in patients with established ASCVD remains uncertain. Fourthly, while cardiac interventions were recorded during follow‐up, the relatively small sample size did not allow (in terms of statistical power) additional subgroup analyses. Fifthly, even though adjustment was implemented for various known confounders, the possibility of residual confounding cannot be completely excluded. Sixthly, our study employed calculated LDL‐C, an approach considered clinically comparable to direct measurement for cardiovascular risk classification due to the absence of proven superiority,^35^, ^36^ though variations in measurement methodologies could affect LDL‐C quantification. The interpretation of attributable risk is contingent upon the assumption of a causal relationship between exposure and outcome. However, due to the observational nature of this study, causal inference cannot be definitively established. Independent replication studies with extended follow‐up periods in diverse populations are necessary to validate the generalizability of these findings beyond the ATTICA study cohort.

## 5 CONCLUSIONS

Our findings suggest that apoB may serve as a valuable biomarker for long‐term ASCVD risk assessment. These results corroborate the prevailing hypothesis that ASCVD risk associated with apoB‐containing lipoproteins is more strongly correlated with the number of apoB particles than with their cholesterol content. Integrating apoB assessment into established risk factor management strategies has the potential to substantially clarify residual ASCVD risk and may lead to more effective treatment approaches, especially in individuals identified as high‐risk based on traditional cholesterol markers.

## AUTHOR CONTRIBUTIONS

Sofia‐Panagiota Giannakopoulou: Conceptualization (Lead), formal analysis (Lead), visualization (Lead), writing—original draft (Lead); Smaragdi Antonopoulou, Evangelos Liberopoulos: Methodology (Equal), writing—reviewing and editing (Lead); Fotios Barkas: Investigation (Equal), writing—reviewing and editing (Equal); Petros P. Sfikakis, Costas Tsioufis: Methodology (Equal), writing—reviewing and editing (Equal); Christina Chrysohoou, Christos Pitsavos: Supervision (Lead), methodology (equal), writing—reviewing and editing (Equal); Demosthenes Panagiotakos: Supervision (Lead), methodology (equal), writing—reviewing and editing (Lead). All authors have read and agreed to the published version of the manuscript.

## FUNDING INFORMATION

The ATTICA study is supported by research grants from the Hellenic Cardiology Society (HCS2002) and the Hellenic Atherosclerosis Society (HAS2003).

## CONFLICT OF INTEREST STATEMENT

The authors declare they have no conflict of interest.

## Data Availability

The data presented in this study are available on request from the corresponding author. The data are not publicly available due to privacy restrictions.
